# Incidence and root causes of surgery cancellations at an academic medical center in Iran: a retrospective cohort study on 29,978 elective surgical cases

**DOI:** 10.1186/s13037-023-00377-6

**Published:** 2023-09-06

**Authors:** Vida Naderi-Boldaji, Mahsa Banifatemi, Raheleh Zandi, Mohammad Hossein Eghbal, Milad Nematollahi, Mohammad Ali Sahmeddini

**Affiliations:** 1 Anesthesiology and Critical Care Research Center, Shiraz University of Medical Sciences, Shiraz, Iran; 2 Namazee Hospital, Shiraz University of Medical Sciences, Shiraz, Iran

**Keywords:** Cancellation, Cases, Elective surgery, Prevalence, Iran

## Abstract

**Introduction:**

Canceling scheduled surgeries on the day of surgery places a heavy burden on healthcare providers and has psychological, social, and financial consequences on patients and their families. This study aimed to investigate the main reasons for cancellations of elective procedures and provide appropriate recommendations to reduce the rate of such avoidable cancellations.

**Methods:**

Data were collected retrospectively from all consecutive elective cases scheduled for various elective surgeries from January 1, 2020 to March 31, 2022 at Namazi Teaching Hospital, a major referral center in southern Iran with a capacity of 938 beds. Daily data were collected on the number of planned electives, cancellations, and reasons for cancellations. Surgical cancellation reasons were categorized as patient-related, surgeon-related, hospital/system-related, and anesthesia-related. Data were expressed as frequency (percentage) and analyzed with SPSS version 19 software.

**Results:**

The cancellation rate on surgery day for elective procedures in all fields was 6.3%. The highest cancellation rate was related to minor surgeries (19%), followed by urology (8%), pediatrics (7%), and plastic surgery (7%). The most common reasons for cancellation were patients not suitable for the procedure (37%), followed by patients who did not follow instructions (10%), lack of time (10.5%), and equipment/supplies problems (10%), and refusal to consent (6%).

**Conclusions:**

According to this study, patients’ unsuitability for surgery, non-compliance with instructions, lack of time, and problems with equipment/supplies are the main reasons for canceling surgery. Proper preoperative assessment and preparation of patients and improved communication between medical teams and patients reduce the cancellation of booked surgeries.

**Supplementary Information:**

The online version contains supplementary material available at 10.1186/s13037-023-00377-6.

## Introduction

Canceling scheduled surgeries on the intended day of surgery is not an uncommon occurrence worldwide. Canceling and delaying surgeries put a huge burden on healthcare providers, including hospitals, physicians, medical assistants, and nursing staff [1], and it has financial, psychological, and social consequences for patients and their families [2–4]. In the United States (US), the value of each wasted minute of operating room time was estimated to be between $10 – $20, and the average canceled operation costs $ 5,000–8,000 [5]. Only one single-center study in Iran has reported the average cost of surgery cancellations to be around US$ 92,049.0 [3].

Long waiting lists cause the migration of pre-scheduled cases to the upper stage, leading to poor patient turnover and lost teaching opportunities in teaching hospitals [6]. Cancellation of elective operations is a parameter to evaluate the quality of patient care and the quality of the management system [7]. The overall elective surgery cancellation rate varies significantly by country and type of setting, ranging from 1 to 30% of booked elective surgeries [8, 9].

The most common reasons for cancellation of elective surgery cases included a wide range of hospital, anesthesiologist, surgeon, and patient-related factors such as bed unavailability due to an increased number of emergency admissions, equipment shortages, ICU bed shortage, inadequate preoperative assessment, not following instructions, etc. [10, 11].

Despite the regional recognition of high-quality healthcare, there has been only one study reviewing the causes of cancellation before and after the implementation of a health sector transformation plan in Iran at Namazi Hospital [3]. This research aimed to investigate the main reasons for elective procedure cancellation and to make appropriate recommendations for reducing the rate of such avoidable cancellations and optimizing the utilization of the workforce and resources.

## Methods

In this retrospective study, all consecutive elective cases scheduled for various elective surgeries from January 1, 2020, to March 31, 2022, were included in Namazi Teaching Hospital. Individuals listed for elective surgery but performed as an emergency before the day of the schedule were excluded from the study. Namazi is the main referral center in southern Iran affiliated with Shiraz University of Medical Sciences, Shiraz, Iran. Also, it serves a large population of neighboring Arab countries of the Persian Gulf. This institution has a total capacity of 938 beds and has maintained an average occupancy rate of 79% during 2021–2022. Patients were clinically evaluated 1–7 days before the surgery in the hospital or outpatient department. Day cases were admitted on the morning of surgery. The list of operations for elective procedures is prepared and sent to the operating rooms by the preceding afternoon. The listed cases are assessed in the ward the evening before surgery, and complicated cases are reported to the consultant anesthetist. Operations data, including the number of electives scheduled, the number of cancellations, and specialty-related reasons for cancellations were collected manually by an operating room nurse. Reasons for surgical cancellations in this study were classified as patient-related, including non-adherence to instructions, refusal to consent, not attending, high blood pressure / uncontrolled blood sugar, upper respiratory tract infections, and impaired cardiac/ pulmonary function; surgeon-related factor that includes patient unsuitable for surgery, change in plan, absence of surgeon, lack of operating theater time; hospital/system-related factors include unavailability of drugs, equipment, and supplies, ICU bed shortage, last-minute emergencies, and anesthesia-related factors that include patient unpreparedness for surgery due to incomplete preoperative assessment. The data were entered into SPSS software version 19 (IBM Co., Armonk, NY, USA), analyzed descriptively, and presented in the tables and text.

## Results

We analyzed 29,978 elective surgical cases scheduled retrospectively; Pediatric and urology surgeries were performed for 7,509 (25.04%) and 6,827 (22.8%) cases in the study period, respectively, the cancellation rate on surgery day for elective procedures in all fields was 6.3% and highest cancellation rate among different surgical subspecialties was related to minor surgeries, for example, intravascular catheter placement, endoscopy, taking a biopsy, etc. (19%), followed by urology (8%), pediatric (7%), and plastic surgeries (7%) (Table 1).

**Table 1:** Total day of surgery cancellation per specialty distribution of booked cases

Specialty	Number of scheduled cases(*n*)	percentage of scheduled cases	Number of cancelled cases (*n*)	Ratio of cancelled/scheduled In each field (%)
**Urology**	6827	22.8	558	8.17
**Pediatric surgery**	7509	25.04	525	6.99
**General surgery**	3506	11.7	203	5.79
**Vascular surgery**	3349	11.17	182	5.43
**Neurosurgery**	2864	9.5	138	4.81
**Thoracic surgery**	1640	5.4	112	6.82
**Plastic surgery**	1165	3.9	80	6.86
**Orthopedics**	1734	5.8	66	3.80
**Gynecological surgery**	1275	4.2	10	0.78
**Minor surgery**	109	0.36	21	19.26
**Total**	29,978	100%	1895	6.32

The highest reasons for cancellation were patient unfit for operation (37%), then failure to follow instructions by the patient (10%), lack of time (10.5%), and equipment/ supplies issues (10%). Other documented reasons (20%) include the patient did not attend, no procedure required, surgeon unavailability, ICU bed shortage, emergency priority, and overscheduled elective surgery (Table 2).

**Table 2:** Reasons for cancellation on day of surgery

reasons	type	frequency	percentage
**Patient unfit for surgery**	PR/AR/SR	701	37
**Patient didn’t follow instruction**	PR	190	10
**Refusal of consent**	PR	114	6
**Patient did not attend**	PR	95	5
**Procedure not required**	PR	95	5
**Lack of time**	SR	198	10.5
**Change in plan**	SR	122	6.5
**Surgeon unavailability**	SR	76	4
**Equipment/ supplies issues**	HR	190	10
**ICU bed shortage**	HR	57	3
**Emergency case priority**	HR	57	3

We reported the details of the cancellation along with the underlying reasons in Table 3.

**Table 3:** Reasons for cancellation on day of surgery-further analysis

Cancellation type	Underlying reason
**Patient unfit for surgery**	Missed clinical work up
	Patient on medication
	Abnormal laboratory result
	High blood pressure / Uncontrolled blood sugar
	Acute medical illness
**Equipment/ supplies issues**	Equipment not available (i.e. intravascular catheter)
	Problems with necessary equipment (i.e. XRAY, echo)

## Discussion

There is considerable variation in the rate of and reasons for cancellations on the day of surgery, depending on the national healthcare service structure and individual hospital policies and management practices. This study aimed to investigate the main reasons for the cancellation of elective procedures and provide appropriate recommendations to reduce the rate of such avoidable cancellations and optimize the use of manpower and resources. The cancellation rate on surgery day for elective procedures in all fields was 6.3%. The highest cancellation rate was related to minor surgeries (19%), followed by urology (8%), pediatrics (7%), and plastic surgery (7%). The most common reasons for cancellation were patients not suitable for the procedure (37%), followed by patients who did not follow instructions (10%), lack of time (10.5%), and equipment/supplies problems (10%), and refusal to consent (6%). Our findings are inconsistent with studies conducted in St. Paul’s Hospital, Addis Ababa (8.9%) [12], Saudi Arabia (7.6%) [13], Brazil (6.8%) [14], and Wales (7.6%) [15], also lower than studies conducted in Sudan (20.2%) [16], Nigeria (20.2%) [17], Germany (12.7%) [18], New Delhi (17.6%) [19], and India (16.49%) [20]. In our research, the rate of cancellation was high in internal surgeries (minor surgery, e.g., intravascular catheter insertion, endoscopy, taking a biopsy, etc. (19%), followed by urology (8%), pediatric (7%), and plastic surgeries (7%). These variations may be explained by the national healthcare service’s structure, individual hospital policies, and management practices. Although there is a well-established pre-assessment service in the study hospital and pre‑anesthetic clinic, the most common reason for cancellation on the surgery day was that the patient was medically unfit for the operation. Some of the reasons identified were disagreement between the outcome at pre-assessment and the opinion of the anesthesiologist in charge on the day of the operation, the deterioration of the patient’s condition between pre-assessment and the operation day, and abnormal laboratory results detected after admission.

Previously, it has been reported that pre-assessment of the patient 30 days before surgery is not associated with a reduction in the volume of cancellations compared to pre-assessment 24 h before surgery [21]. If the health status of patients is assessed too early before surgery, it may change in the period leading up to surgery. In addition, if the patient is diagnosed late for proper surgery, the time available for any intervention will be limited, and there will not be enough time to make appropriate changes in the surgery list. Thus, the effectiveness of providing surgical services will be compromised [22].

The second most common reason for cancellation on surgery day was lack of time at the study hospital. Published studies from public hospitals have reported similar rates of cancellations on surgery days due to lack of time [23, 24]. Major surgeries in all specialties are performed in the study center, which sometimes requires the simultaneous attendance of several surgical specialists. These long-term surgeries disrupt the process of performing other surgeries as well.

Another common reason for cancellation on surgery’s day in this hospital was that patients did not follow guidelines, consistent with studies conducted worldwide [25, 26].

Patients turn up for their operations without following the fasting instructions or eliminating their anticoagulation medications, leading to delays, rescheduling, or cancellations. The root cause of patient noncompliance with preoperative instructions is a multifactorial and arduous problem to manage. It is possible that the surgeon did not express the specific fasting instructions and instructions for taking or removing their current medications understandably, or the patients did not remember the instructions well because of impaired memory [26]. Instructions should be given verbally, information sheets should be provided, instructions should be repeated in pre-assessment clinics, and information should be shared with all those involved in the patient’s care, such as ward staff in inpatient cases or patients relevant in day cases. Also, sending automated reminders can prepare patients for surgery and prevent cancellations. The last common reason for cancellation at the study hospital was Equipment/ supplies issues.

The limited capacity to provide basic medical equipment to diagnose and treat all referring patients can be partly attributed to the indirect effects of the Health Sector Transformation Plan (HSEP), which was carried out in hospitals affiliated with the Ministry of Health and Medical Education (MOHME), including this hospital, to reduce catastrophic health expenses (CHE) to 1% as announced in the fifth plan of economic, social and cultural development. As a result, the amount paid by patients eligible for basic health insurance will be reduced by 6% and 3% of the total hospitalization costs, and all people without basic health insurance will be covered for free [27]. In this situation, the high load of patients referring to public hospitals to use these services, the limited financial resources of the hospital, and the failure to return the hospital expenses on time by the insurance organizations reduce the ability of the hospital to provide the necessary equipment.

## Limitations

This study was a retrospective chart review of the main reasons for canceling elective procedures in the largest referral center in southern Iran. It was so hard to separate reasons for cancellations into distinct categories; some cancellation reasons may fall into more than one category. We are interested in preparing a special questionnaire for canceled surgeries in which the reason for the cancellation is clearly specified.

## Conclusion

This study highlights some extent the causes of cancellation of operations are avoidable. Patients not being suitable for the procedure, not following instructions, lack of time, and equipment/supplies issues are the main reasons for cancellation.

Although automatic reminders are helpful for patients who do not adhere to the preoperative instructions, how well patients understand preoperative instructions and the cooperation of all involved in patient care are unavoidable factors.

Determining the exact duration of each surgery is difficult, but setting a controlled schedule with the cooperation and advice of the surgical and anesthesia teams based on their experience seems to prevent overbooking of the operating room list and reduce the cancellation rate in university hospital settings.

To reduce the hospital’s limitations in providing medical equipment, it seems that our policy in the Health Sector Transformation Plan (HSEP) and obligations of insurance organizations to return hospital expenses on time need revision.

### Electronic supplementary material

Below is the link to the electronic supplementary material.

Supplementary Material 1

## Data Availability

Data related to the study are available upon reasonable request to the corresponding author.
